# Makelabels: a Bash script for generating data matrix codes for collection management

**DOI:** 10.3897/BDJ.4.e9583

**Published:** 2016-08-01

**Authors:** Theresa A. Dellinger, Victoria Wong, Paul E. Marek

**Affiliations:** 1 Virginia Tech, Blacksburg, United States of America Virginia Tech Blacksburg United States of America

**Keywords:** collections management, curation, data matrix barcode, digitization, entomology collection, natural history collection, open source

## Abstract

**Background:**

Digitization of natural history collections allows easy access and reuse of the invaluable biodiversity data held within a collection by providing access to specimen level data through the Internet. Each digitized specimen in a database requires a unique catalog number to distinguish it from the many other biologically unique specimens within the collection. However, there are few open source barcode generators available, and of these even fewer platforms exist to enable the mass production of barcode labels required by natural history collections. We developed a low-cost, open source solution to generating data matrix barcodes with unique catalog numbers for use in the Virginia Tech Insect Collection.

**New information:**

Here we describe the *makelabels* script, which uses the open source Unix packages *libdmtx* and *ImageMagick* to generate unique specimen labels containing both a human-readable catalog number and a machine-readable data matrix barcode. The mass production of labels and use of both types of catalog symbology provides flexibility in specimen management and increased efficiency in digitization and specimen processing workflows.

## Introduction

Natural history collections (NHCs) are invaluable repositories of biological information documenting the biodiversity of our planet (Winker 2004). Increasingly, NHCs have proven useful in providing evidence of climate change, pollution, species decline, habitat loss, and threats to agriculture and public health (e.g.: Colla et al. 2012, Graham et al. 2004, Lavoie 2013, Lister 2011, Page et al. 2015, Pyke and Ehrlich 2010, Suarez and Tsutsui 2004, Winker 2004).

One force driving the acceleration of interest in NHCs is enhanced accessibility. In the past, using a biological collection has been limited to: physically visiting a museum’s holdings; requesting a loan of material; or less often, obtaining a spreadsheet or email detailing the label data associated with a specimen. These traditional means of access created an unintentional bottleneck where only a limited number of people were privy to the specimens and their associated data. Even if a museum had developed a catalog, frequently the included information was not very extensive and often limited to a species list that lacked specimen-level data. In contrast, digitization of a NHC serves specimen photographs, a database of associated label data (collection localities, coordinates, dates of collection, habitat notes, etc.), and georeferenced distribution maps online—where these data can be read, retrieved, and reused by anyone with an Internet connection. Complementary data such as field journal entries made by collectors or audio recordings of species’ songs can be added to the entry for a specimen. This approach places a wealth of previously inaccessible “dark” information into the hands of all interested parties (Page et al. 2015). Natural history collections and their data are on the forefront of biological research, and we are just now glimpsing the power and capabilities of this multidimensional source of biological data (Baird 2010, Scoble 2010, Suarez and Tsutsui 2004, Vollmar et al. 2010).

Researchers are using these digitized databases to answer a wide range of important questions addressing: ecological processes and the biodiversity of species (e.g., Nelson et al. 2012, Newbold 2010, Ward 2012), pollinator decline (Colla et al. 2012,) threats to agriculture (Sánchez-Cordero and Martínez-Meyer 2000), phenological changes due to climate change (Fenberg et al. 2016), and risk assessment of invasive species (Peterson and Vieglais 2001). Interagency working groups have prioritized resources for building support for these data sources, such as the Network Integrated Biocollections Alliance, Interagency Working Group on Scientific Collections, and National Science Foundation (Collections in Support of Biological Research, Advancing Digitization of Biodiversity Collections, and Postdoctoral Research Fellowships using Biological Collections). Moreover, to address the growing need for digitized NHC data directly relevant to human health, a renewed focus to establish a vector-specific database of specimen data, infectious disease agents, and patient samples has been proposed to combat emerging infectious diseases across the globe (DiEuliis et al. 2016).

A useful model of the digitization process has been described by Nelson et al. (2012). In brief, each specimen is photographed with its physical labels, the resulting image is processed and uploaded to a relational database, information associated with that specimen is entered into the database, and the locality information for the specimen is georeferenced as well. To avoid confusion among individuals, each specimen must be labeled with a unique catalog number that is used to access its records in the database. Unique identifiers are critical to managing and understanding biological variation because two individuals possess distinct properties, and can be genetically distinct, phenotypically unique, and potentially even separate morphologically cryptic species. Unique identifiers are essential to preserve identity, handle biological variation, and for repeatable science. A lack of unique identifiers is most noticeable, for example, when published species identifications are called into question and specific material is unable to be tracked unequivocally to material deposited in a NHC for re-identification (Turney et al. 2015). A unique identifier is often printed as an alphanumeric code on the specimen label, although the identifier may also be a two-dimensional barcode that can be scanned by an optical reader to ensure transcription fidelity (iDigBio. 2016). A data matrix barcode offers the benefit of storing large amounts of information in a relatively small graphic, an important feature when label size is constrained by storage space.

The Virginia Tech Insect Collection (VTEC) has begun digitizing its specimen data online to serve a wider audience. A member of the Symbiota Collections of Arthopods Network (SCAN, Gries et al. 2014), VTEC’s data are in the public domain (Creative Commons CCZero) to allow unimpeded reuse of the data (VPI-VTEC in SCAN). Dating to 1888, VTEC holds nearly a half-million specimens largely collected from the mid-Atlantic and with a strong focus on the biodiverse Appalachian region. The collection is the official repository for specimens in the state of Virginia and represents a rich insect diversity, including pollinators and many native species whose populations are in decline due to habitat loss. Appalachia is a topographically complex and ancient region with an exceptional repository of high species richness and relative rarity of taxa (Stein et al. 2000). Many species in the region are short-range endemics with a critically imperiled G1 conservation status as ranked by NatureServe (Master et al. 2012), representing irreplaceable biodiversity with strong conservation value to the state and nation. The specimen data found in VTEC encompasses much of this unique biodiversity and is an underutilized resource that could benefit research, extension, and teaching at the state, regional, and national stages.

However, like many NHCs, VTEC faces limited funding that impacts basic curation and management of the collection. In the interest of conserving funding as much as possible, and to share an open source resource with other collections, we wrote our own script to generate labels with unique specimen identifiers as a low-cost alternative to commercially available software or costly pre-printed labels. The VTEC uses 9-digit locally unique identifiers printed on labels and 36-digit universally unique identifiers (UUIDs) automatically assigned and stored in SCAN for specimen management. Here we describe a script we developed using open source libraries and a graphics package for creating specimen labels with a unique alphanumeric reference number that is also encoded in a data matrix barcode.

## Project description

### Design description

**Figure 1.**
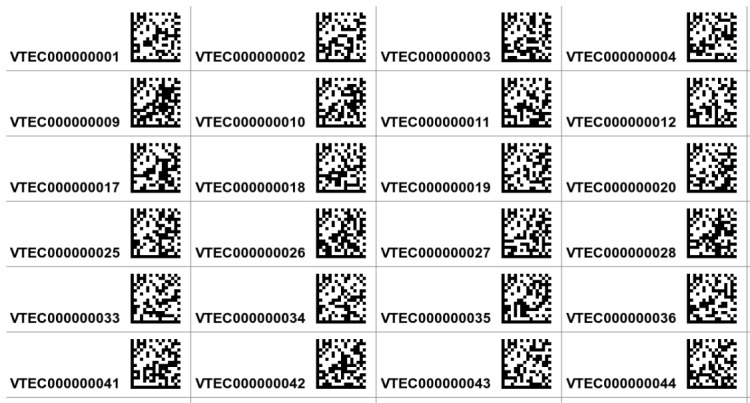
Labels generated by the *makelabels* script, including the unique accession number and the data matrix code returning the unique number when scanned.

We developed a Bash shell script, *makelabels*, which uses readily-available open source software packages to create labels for specimens in the VTEC. Each label consists of a locally unique number and a corresponding data matrix barcode for each specimen (Fig. 1). This reference number uses a “VTEC” prefix to denote the collection’s name and a 9-digit number for the specimen’s database catalog. The data matrix barcode returns the VTEC specimen number when scanned with an optical reader. We chose a data matrix barcode instead of a quick response (QR) code or one-dimensional universal product code (UPC) symbology because it is functional at a much smaller size, retains error correction (data can be retrieved even if a cell is damaged or missing), and stores up to 2,335 alphanumeric characters. Two-dimensional size is important to us because labels are pinned beneath specimens and reduction of an individual specimen footprint saves a considerable amount of space in the collection drawers.

We used runnable programs from the two open source packages, *libdmtx* 0.7.4 (Laughton 2016) and *ImageMagick* 6.9.3-6 (ImageMagick Studio, LLC. 2016). The *dmtxwrite* program from the *libdmtx* distribution was used to generate one data matrix barcode at a time, and the *ImageMagick* commands *convert* and *composite* were used to merge and assemble barcode images into a printable page. The *ImageMagick* commands also produced the alphanumeric VTEC reference number and a grid to facilitate cutting the labels.

When executed, *makelabels* first creates a blank image to contain a page of labels and adds the lines that make up the grid. The program *libdmtx* generates each barcode into a temporary file and then *makelabels* merges the barcode and the alphanumeric VTEC number into the page image using the *ImageMagick* composite command. Using individual commands and intermediate files with *makelabels* allows for easy modification of the label appearance as needed and is scalable to use UUIDs longer than our 13-alphanumeric text.

Including both the symbology of the barcode and the alphanumeric code on a label allows for maximum flexibility as the label can be read by a person or an optical reader. The resulting labels are easily read by a number of free barcode scanner applications available for iOS and Android operating systems, eliminating the need to purchase a commercial optical reader.

A README.txt file accompanies the Bash script online at Github and describes the process of using *makelabels*. The printable page is encoded as a PNG file with letter-sized dimensions (8.5 X 11 inches or 216 X 279.4 mm). While printing preferences vary according to institution and conservation protocol, we use an Epson Stylus C88 inkjet printer with black DURABrite pigment-based ink (Epson America, Inc., Long Beach, CA). We print the labels on cotton archival 32 lb. paper using the photo quality mode of the printer to maintain anti-aliasing and resolution. Labels are cut by hand, according to the grid marks on the printed label page.

### Funding

This project is supported by a NSF Collections in Support Biological Research (CSBR) award, DBI #1458045.

## Web location (URIs)

Download page: https://zenodo.org/record/58868#.V5oL_5MrJBw

## Technical specification

Programming language: Bash shell script

Operational system: OS/X and Linux

Interface language: English

## Repository

Type: Git

Browse URI: https://github.com/apheloria/makelabels

Location: DOI: 10.﻿5281/zenodo.58868

## Usage rights

### Use license

Creative Commons Public Domain Waiver (CC-Zero)

## Additional information

### Use and customization

The script is fully documented and includes configuration parameters in README.txt. As written, a user can edit the script to change the overall size of the cell, which includes the alphanumeric VTEC code, the data matrix barcode, and the placement of the VTEC code and data matrix barcode within the cell. Font style and size can also be changed as needed. The complete path for a font is needed if the code is run in Linux.

Although the *makelabels* script is recommended for use on a Unix operating system, a Windows system may be used with a Unix-like command-line interface such as *Cygwin* (Red Hat, Inc. 2016) to provide functionality for the script. Alternatively, one could run Linux under Windows using a machine emulator such as the *VirtualBox* (Oracle Corporation 2016) platform package.

Care should be taken when adjusting some of the configuration parameters. For example, the resolution is set to work with *ImageMagick* commands. Cell X and Y margins are defined in pixels to avoid handling problems with some printers, but changing the resolution will alter the pixel description and the overall appearance of the labels.

When initiating the software, the user can specify the beginning and ending sequence numbers to be printed on the labels. If no end number is provided, the code will stop after generating a total of 26 labels (start number plus 25 additional labels). A printer name can be specified as well for immediate printing. Otherwise, labels will be generated and saved as PNG files (*e.g.*, page1.png, page2.png, …). Because the user specifies the beginning and ending number for each batch of labels, the user must keep track of which labels have been created previously to avoid duplicates.

The disadvantage of *makelabels* is that the file operations are assembled into a Bash script that is relatively inefficient and slow when compared to compiled programs. Now that the steps to use *dmtxwrite* and the commands in *ImageMagick* are known, a compiled program could be written using the *libdmtx* library to create a page of labels in memory and without using intermediate files in each run.

While we chose to write labels encoding only the unique VTEC specimen number, the *makelabels* script can be modified to include more information, such as a URL to a database containing a specimen record, and up to 2,335 alphanumeric numbers (approximately two-thirds of a page of text in 12-point font). Additionally, some barcode scanning applications will scan, track, and automatically export coded information into a file. This would obviate the need to manually enter each unique code into a database to pull the associated information for that item.

### Broader applications

**Figure 2.**
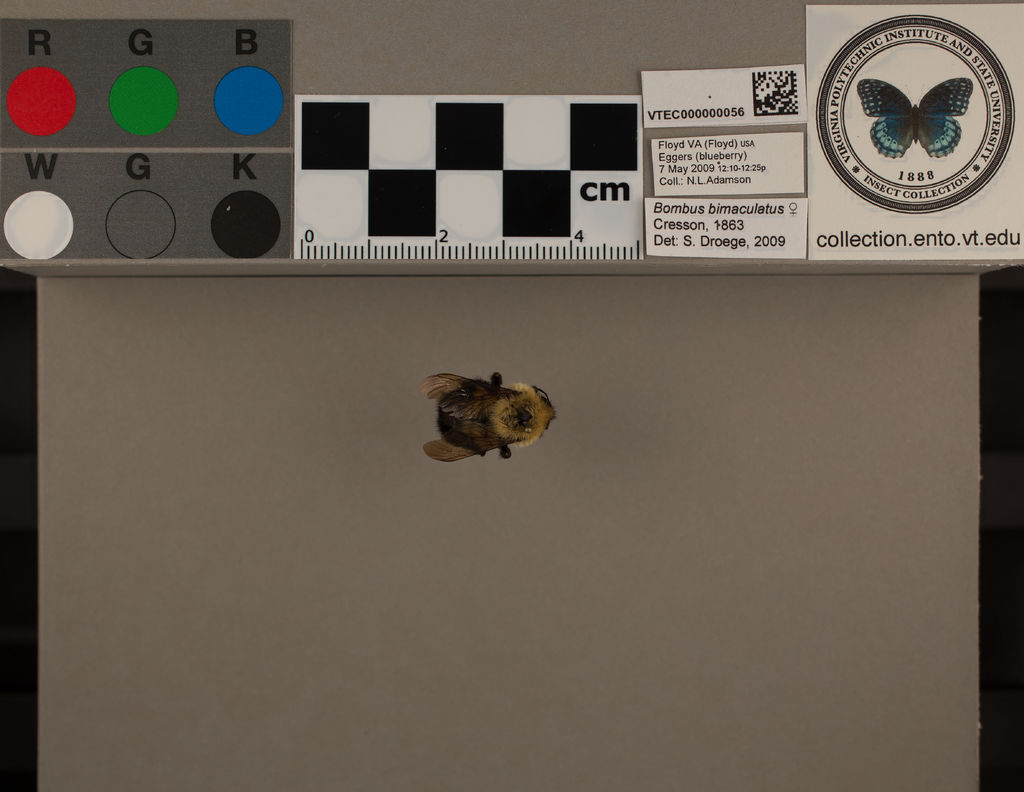
Photo of two-spotted bumble bee, *Bombusbimaculatus*, with data matrix barcode label, as prepared for the digitization project in the Virginia Tech Insect Collection (VTEC).

The concept of a natural history collection has expanded over time from whole, preserved specimens to include other biological collections, such as living stocks and cultures, and preserved tissues. Our labels can be used with these types of collections, and also as part of collection management in geology, paleontology, archeology, and related fields. For our purposes, the data matrix barcoded labels created by *makelabels* are an integral part of digitizing the VTEC collection (Fig. 2). However, these scannable labels can be used within a lab to quickly identify the contents of storage units, such as unit trays, and larger organizational units such as cabinets. We plan to label our Cornell insect drawers, which house individual specimens, using the same process but with larger data matrix barcodes positioned on the front of the drawer.
